# Synthesis of New Riminophenazines with Pyrimidine and Pyrazine Substitution at the 2-N Position

**DOI:** 10.3390/molecules16086985

**Published:** 2011-08-16

**Authors:** Gang Zhang, Hao Zhang, Xiaojian Wang, Chun Li, Haihong Huang, Dali Yin

**Affiliations:** Department of Medicinal Chemistry, Beijing Key Laboratory of Active Substance Discovery and Drugability Evaluation, Institute of Materia Medica, Peking Union Medical College and Chinese Academy of Medical Sciences, 1 Xian Nong Tan Street, Beijing 100050, China

**Keywords:** riminophenazine, N-arylation, palladium catalyst

## Abstract

New riminophenazines with pyrimidine and pyrazine substituents at the 2-position were successfully synthesized. The key step is the 2-N-arylation of riminophenazines with pyrimidine and pyrazine. The optimized reaction conditions involve the use of a Pd_2_(dba)_3_/DPPF/Cs_2_CO_3_/toluene combination.

## 1. Introduction

**Figure 1:**
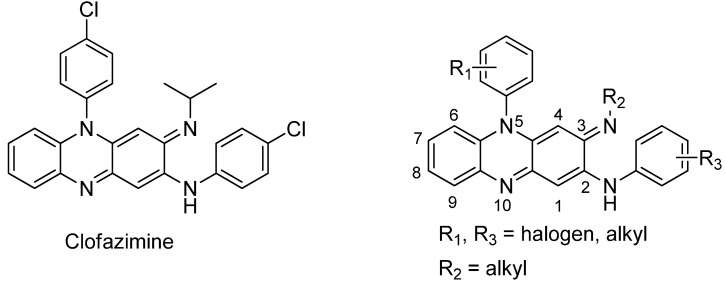
Chemical structures of clofazimine and known riminophenazine derivatives.

Riminophenazines such as clofazimine (Figure 1) are of great interest to many medicinal chemists due to their various biological activities in the anti-mycobacterial [1,2], anti-tumor [3] and anti-inflammatory areas [4,5,6], especially for multidrug resistant tuberculosis (MDR-TB) [7]. In our ongoing search for novel antituberculotic riminophenazines, a series of new compounds were designed, including riminophenazines with heteroaromatic substituents at the 2-N position.

There are generally two methods for the preparation of riminophenazines. Condensation of two molecules of *N*-chlorophenyl-1,2-diaminobenzene by oxidation with FeCl_3_ to form the tricyclic core of a riminophenazine gives the same substituents on the 2-N-phenyl and 5-phenyl rings [8]. For the compounds with different substituents in the 2-N and 5 positions, a method developed by Andre Girard [9] allows step by step introduction of different substituted anilines into the molecules. The synthesis of riminophenazine with peripheral heteroaromatic substituents in the molecule has not been reported. Herein we report the synthesis of new riminophenazines with pyrimidine and pyrazine substitutions at the 2-N position.

## 2. Results and Discussion

**Scheme 1:**
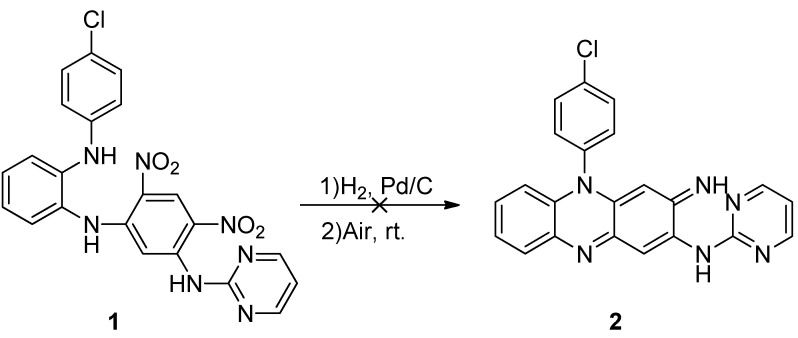
Cyclization of riminophenazine core with pyrimidine substituent.

An initial investigation of the synthesis of target compounds followed known procedures [9] (Scheme 1). Unfortunately, the key intermediate **1** could not be converted into the desired iminophenazine **2**. Only a black tar was obtained, possibly due to instability of the intermediates with pyrimidine substituents.

**Scheme 2:**
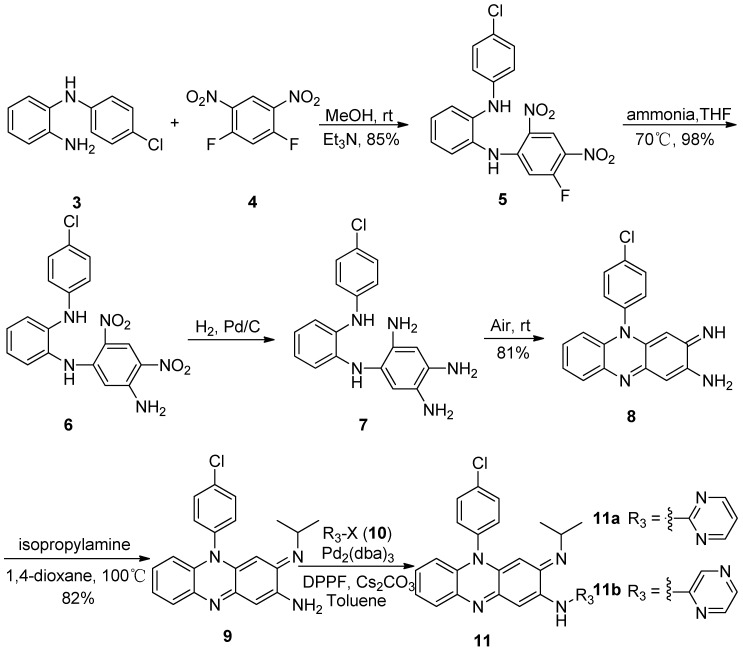
New synthetic route for riminophenazines with heteroaromatic substituents.

To overcome the difficulties in the key cyclization step of the synthesis, the synthetic procedures were modified and the attachment of heteroaromatic substituents was moved to the last step of the synthesis to avoid decomposition of the intermediates Scheme 2). Thus, the synthesis was started from 1,5-difluoro-2,4-dinitrobenzene (**4**), which is more reactive towards amine substitution. The two fluoro groups of compound **4** was subsequently replaced by *N*-(4-chlorophenyl)benzenediamine (**3**) and ammonia to give the key intermediate **6**. The dinitro groups of **6** were reduced either by catalytic hydrogenation over Pd-C or by zinc powder reduction and the intermediate **7**, without isolation, was exposed to air and cyclized to form the desired riminophenazin core **8** with an amino group pending in the 2-position. Compound **9** was obtained by displacement of the imino moiety with isopropylamine in a sealed bomb. When the palladium catalyzed *N*-arylation, better known as the Buchwald-Hartwig reaction [10], was applied to the 2-N arylation of **9** with 2-bromopyrimidine under the usual reaction conditions, none of the desired product was formed. After screening of condition variables, such as Pd catalyst, ligand, base, and solvent, a combination suitable for the *N*-arylation of **9** with 2-bromo-pyrimidine was found (Entry 7). The target compound **11a** was thus obtained in 95.8% yield.

When the optimized combination was used for the coupling of **9** with 2-bromopyrazine, the desired product **11b** was also obtained in 95.2% yield.

**Table 1:** Optimization of *N*-arylation of riminophenazines ^a,b^. 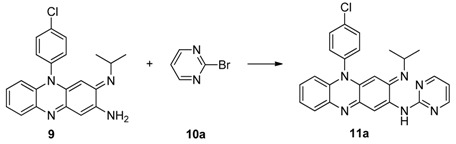

Entry	Catalyst	Ligand	Base	Solvent	T(°C)/Time(h)	Yield(%) ^c^
1 [11]	Pd_2_(dba)_3_	IPr·HCl ^d^	t-BuOK	1,4-dioxane	100/24	0
2 [12]	PdCl_2_	P(o-tolyl)_3_	t-BuOK	xylene	110/24	0
3	PdCl_2_	DPPF	Cs_2_CO_3_	toluene	110/8	57.6
4	Pd(OAc)_2_	DPPF	Cs_2_CO_3_	THF	68/8	0
5	Pd(OAc)_2_	DPPF	Cs_2_CO_3_	toluene	70/24	25.1
6	Pd(OAc)_2_	DPPF	Cs_2_CO_3_	toluene	110/8	31.3
7	Pd_2_(dba)_3_	DPPF	Cs_2_CO_3_	toluene	110/2	95.8
8 [13]	Pd_2_(dba)_3_	*rac*-BINAP	Cs_2_CO_3_	toluene	110/12	88.9

^a^ Reaction conditions: **9** (1.0 equiv, 0.20mmol), 2-bromopyrimidine (1.5 equiv), catalyst (2 mol %), ligand (8 mol %), base (1.5 equiv); ^b^ The product were purified by flash silica gel chromatography; ^c^ All yields are reported for isolated and purified products; ^d^ IPr•HCl = 1,3-bis(2,6-diisopropylphenyl)-imidazolium chloride.

## 3. Experimental

### 3.1. General

All chemicals and reagents were purchased from commercial sources and used without further purification. All reactions were monitored by TLC. Chromatography refers to open column chromatography (CC) on silica gel (SiO_2_; 160–200 mesh). Melting points were determined on a Yanaco micrometer and are uncorrected. ^1^H- and ^13^C-NMR spectra were recorded on Varian Mercury 300 MHz and 400 MHz spectrometers; chemical shifts δ in ppm rel. to TMS or DMSO-*d*_6_ as internal standards. High resolution mass spectra were recorded on an Agilent LC/MSD TOF spectrometer.

*N-(4-Chlorophenyl)-N’-(5-fluoro-2,4-dinitrophenyl)benzene-1,2-diamine* (**5**): 2-(4-Chloroanilino)-aniline (20 mmol) and triethylamine (3 mL) were added to a solution of 1,5-difluoro-2,4-dinitrobenzene (20 mmol) in methanol (100 mL). The reaction mixture was stirred at rt for 4 h, then filtered, and the cake was washed with methanol and dried to give compound **5** in 85% yield. mp: 209–211 °C, ^1^H-NMR (300 MHz, DMSO-*d*_6_): δ 9.97 (s, 1H), 8.88 (d, *J* = 8.1 Hz, 1H), 7.89 (s, 1H), 7.31 (m, 3H), 7.22 (d, *J* = 8.1 Hz, 2H), 7.06 (m, 1H), 6.97 (d, *J* = 8.1 Hz, 2H), 6.47 (d, *J* = 14.1 Hz, 1H); ^13^C-NMR (100 MHz, DMSO-*d_6_*) *δ*: 158.6 (d, *J* = 249.9 Hz), 148.2, 148.1, 141.7, 139.1, 128.8, 128.2, 126.8, 126.4, 125.8, 125.7, 123.9, 121.6, 119.5, 118.8, 103.1 (d, *J* = 27.4 Hz); HRMS (ESI-TOF^+^): m/z [M+H]^+^ calcd. for C_18_H_13_ClFN_4_O_4_: 403.0604; found: 403.0608.

*N-(5-Amino-2,4-dinitrophenyl)-N’-(4-chlorophenyl)benzene-1,2-diamine* (**6**): A mixture of compound **5** (15 mmol), 25% ammonia (10 mL) and THF (20 mL) was heated to 70 °C for 12 h in a sealed bomb, then about 20 mL of THF was removed under reduced pressure. After being cooled to rt. the suspension was filtered, and the cake was washed with CH_2_Cl_2_ to give compound **6** in 98% yield. mp: 248–249 °C, ^1^H-NMR (300 MHz, DMSO-*d*_6_): δ 8.92 (s, 1H), 7.77 (m, 3H), 7.28 (m, 5H), 7.06 (m, 1H), 7.00 (m, 2H), 6.15 (s, 1H), 5.76 (s, 1H); ^13^C-NMR (100 MHz, DMSO-*d_6_*) *δ*: 149.6, 146.3, 144.3, 142.4, 138.9, 128.7, 127.9, 127.8, 127.6, 125.0, 123.9, 123.6, 121.7, 119.3, 118.9, 96.8; HRMS (ESI-TOF^+^): m/z [M+H]^+^ calcd. for C_18_H_15_ClN_5_O_4_: 400.0807; found: 400.0806.

*5-(4-Chlorophenyl)-3-imino-3,5-dihydrophenazin-2-amine* (**8**): Compound **6** (10 mmol) was suspended in anhydrous methanol (100 mL). The mixture was hydrogenated at 40 psi over 10% Pd-C (0.4 g). Then the Pd-C was removed by filtration. The filtrate containing compound **7** was stirred at rt in contact with air overnight. The solid formed was filtered, washed with methanol, and dried to give compound **8** in 81% yield. mp: 238–240 °C, ^1^H-NMR (300 MHz, DMSO-*d*_6_): δ 7.86 (d, *J* = 8.7 Hz, 2H), 7.76 (m, 1H), 7.59 (d, *J* = 8.7 Hz, 2H), 7.31 (m, 2H), 6.78 (brs, 2H), 6.57 (m, 1H), 6.49 (s, 1H), 5.45 (s, 1H); ^13^C-NMR (100 MHz, DMSO-*d_6_*) *δ*: 149.8, 149.3, 148.1, 136.0, 135.6, 134.5, 133.2, 131.4, 130.8, 130.5, 128.2, 127.7, 123.2, 114.3, 99.8, 96.0; HRMS (ESI-TOF^+^): m/z [M+H]^+^ calcd. for C_18_H_14_ClN_4_: 321.0902; found: 321.0894.

*5-(4-Chlorophenyl)-3-(isopropylimino)-3,5-dihydrophenazin-2-aminee* (**9**): Compound **8** (5 mmol), isopropylamine (25 mmol) and dioxane (100 mL) were mixed in a sealed bomb and stirred at 110 °C for 24 h. After being cooled to rt, water was added, and the mixture was suction filtered, washed with water and dried to give a dark solid. The solid was purified by flash column chromatography (EtOAc/hexane 1:2) to give compound **9** in 82% yield. mp: 234–235 °C, ^1^H-NMR (300 MHz, DMSO-*d*_6_): δ 7.84 (d, *J* = 8.4 Hz, 2H), 7.59 (m, 1H), 7.55 (d, *J* = 8.4 Hz, 2H), 7.14 (m, 2H), 6.39 (m, 3H), 6.27 (s, 1H), 5.11 (s, 1H), 3.29 (m, 1H), 0.98 (d, *J* = 6.3 Hz, 6H); ^13^C-NMR (100 MHz, DMSO-*d_6_*) *δ*: 150.7, 150.3, 149.5, 136.2, 135.3, 134.2, 133.9, 131.5, 131.0, 130.7, 127.2, 126.6, 122.4, 113.8, 97.9, 88.2, 48.8, 23.4; HRMS (ESI-TOF^+^): m/z [M+H]^+^ calcd. for C_21_H_2__0_ClN_4_: 363.1371; found: 363.1367.

*5-(4-Chlorophenyl)-3-(isopropylimino)-N-pyrimidin-2-yl-3,5-dihydrophenazin-2-amine* (**11a**): Under an atmosphere of N_2_, toluene (50 mL), 2-bromopyrimidine (3 mmol), compound **9** (2 mmol), Pd_2_(dba)_3_ (0.1 mmol), DPPF (0.4 mmol) and Cs_2_CO_3_ (3 mmol) were subsequently added to a two-necked round-bottomed flask with a reflux condenser. The mixture was refluxed for 2 h, allowed to cool to rt, and filtered. The filtrate was concentrated under reduced pressure and the residue was purified by flash silica gel chromatography (EtOAc/hexane 1:2) to give compound **11a** in 95.8% yield. mp: 232–235 °C, ^1^H-NMR (300 MHz, CDCl_3_) *δ*: 9.76 (1H, br s), 8.56 (2H, d, *J* = 4.5 Hz), 8.49 (s, 1H), 7.77 (1H, d, *J* = 6.9 Hz), 7.71 (2H, m), 7.32 (2H, m), 7.17 (2H, m), 6.83 (1H, m), 6.44 (1H, d, *J* = 6.9 Hz), 5.28 (1H, s), 3.47 (1H, m), 1.10 (6H, d, *J* = 6.0 Hz); ^13^C-NMR (100 MHz, CDCl_3_) *δ*: 159.3, 157.9, 151.8, 150.1, 140.2, 136.0, 135.7, 135.0, 132.0, 131.7, 130.5, 128.8, 128.3, 122.8, 113.7, 113.5, 108.4, 89.0, 49.4, 23.6; HRMS (ESI-TOF^+^): m/z [M+H]^+^ calcd. for C_25_H_22_ClN_6_: 441.1589; found: 441.1585.

*5-(4-Chlorophenyl)-3-(isopropylimino)-N-pyrazin-2-yl-3,5-dihydrophenazin-2-amine* (**11b**): Under an atmosphere of N_2_, toluene (50 mL), 2-bromopyrazine (3 mmol), compound **9** (2 mmol), Pd_2_(dba)_3_ (0.1 mmol), DPPF (0.4 mmol) and Cs_2_CO_3_ (3 mmol) were added in turn to a two-necked round-bottomed flask with a reflux condenser. The mixture was refluxed for 2 h, allowed to cool, and filtered. The filtrate was concentrated under reduced pressure and the residue was purified by flash silica gel chromatography (EtOAc/hexane 1:2) to give compound **11b** in 95.2% yield. mp: 228–230 °C, ^1^H-NMR (300 MHz, CDCl_3_) *δ*: 8.43 (1H, s), 8.29 (2H, m), 8.05 (1H, d, *J* = 2.4 Hz), 7.77 (1H, d, *J* = 6.9 Hz), 7.71 (2H, d, *J* = 8.1 Hz), 7.31 (2H, d, *J* = 8.1 Hz), 7.17 (2H, m), 6.45 (1H, d, *J* = 7.8 Hz), 5.28 (1H, s), 3.47 (1H, m), 1.11 (6H, d, *J* = 6.3 Hz); ^13^C-NMR (100 MHz, CDCl_3_) *δ*: 151.4, 151.0, 150.4, 141.6, 140.0, 136.4, 135.9, 135.8, 135.5, 135.1, 131.7, 130.4, 128.9, 128.4, 123.0, 113.8, 107.5, 88.9, 49.3, 23.6; HRMS (ESI-TOF^+^): m/z [M+H]^+^ calcd. for C_25_H_22_ClN_6_: 441.1589; found: 441.1581.

## 4. Conclusions

In summary, we have successfully synthesized riminophenazines with a pyrimidine or pyrazine substituent on the molecule. To our knowledge, this is the first synthesis of heteroaromatic-substituted riminophenazine derivatives and it should prove valuable for the study of the chemistry and biological activity of this class of compounds. 

## References

[1] Barry V.C., Belton J.G., Conalty M.L., Twomey D. (1948). Anti-tubercular activity of oxidation products of substituted o-phenylene diamines. Nature.

[2] Browne S.G., Hogerzeil L.M. (1962). B663 in the treatment of leprosy. Preliminary report of a pilot trial. Lepr. Rev..

[3] van Rensburg C.E., van Staden A.M., Anderson R. (1993). The riminophenazine agents clofazimine and B669 inhibit the proliferation of cancer cell lines *in vitro* by phospholipase A2-mediated oxidative and nonoxidative mechanisms. Cancer Res..

[4] Imkamp F.M. (1968). A treatment of corticosteroid-dependent lepromatous patient in persistent erythema nodosum leprosum: A clinical evaluation of G.30320 (B663). Lepr. Rev..

[5] Mackey J.P., Barnes J. (1974). Clofazimine in the treatment of discoid lupus erythematosus. Brit. J. Dermatol..

[6] Kaplan B., Trau H., Sofer E., Feinstein A., Schewach-Millet M. (1992). Treatment of pyoderma gangrenosum with clofazimine. Int. J. Dermatol..

[7] Yew W.W., Chau C.H. (1996). New antimycobacterial agents. Monaldi Arch. Chest Dis..

[8] Barry V.C., Belton J.G., O’Sullivan J.F., Twomey D. (1956). The oxidation of derivatives of o-phenylenediamine. Part I. Isomeric phenazine pigments obtained by oxidation of 2-aminodiphenylamine hydrochloride. J. Chem. Soc. (Resumed).

[9] Girard A., Ray A. (1970). Novel phenazine compounds and process.. U.S. Patent 3,499,899.

[10] Vo G.D., Hartwig J.F. (2009). Palladium-catalyzed coupling of ammonia with aryl chlorides, bromides, iodides, and sulfonates: A general method for the preparation of primary arylamines.. J. Am. Chem. Soc..

[11] Grasa G.A., Viciu M.S., Huang J., Nolan S.P. (2001). Amination reactions of aryl halides with nitrogen-containing reagents mediated by Palladium/Imidazolium salt systems. J. Org. Chem..

[12] Guram A., Rennels R., Buchwald S. (1995). A simple catalytic method for the conversion of aryl bromides to arylamines. Angew. Chem. Int. Ed. Engl..

[13] Tietze M., Iglesias A., Merisor E., Conrad J., Klaiber I., Beifuss U. (2005). Efficient methods for the synthesis of 2-hydroxyphenazine based on the Pd-catalyzed *N*-arylation of aryl bromides. Org. Lett..

